# Simulation of Thermal and Electric Field Distribution in Packaged Sausages Heated in a Stationary Versus a Rotating Microwave Oven

**DOI:** 10.3390/foods10071622

**Published:** 2021-07-13

**Authors:** Wipawee Tepnatim, Witchuda Daud, Pitiya Kamonpatana

**Affiliations:** 1 Department of Food Science and Technology, Faculty of Agro-Industry, Kasetsart University, Bangkok 10900, Thailand; wipawee.tep@ku.th; 2 National Metal and Materials Technology Center, National Science and Technology Development Agency, Thailand Science Park, Pathum Thani 12120, Thailand; witchuds@mtec.or.th

**Keywords:** microwave heating, mathematical modeling, simulation, rotation, sausages

## Abstract

The microwave oven has become a standard appliance to reheat or cook meals in households and convenience stores. However, the main problem of microwave heating is the non-uniform temperature distribution, which may affect food quality and health safety. A three-dimensional mathematical model was developed to simulate the temperature distribution of four ready-to-eat sausages in a plastic package in a stationary versus a rotating microwave oven, and the model was validated experimentally. COMSOL software was applied to predict sausage temperatures at different orientations for the stationary microwave model, whereas COMSOL and COMSOL in combination with MATLAB software were used for a rotating microwave model. A sausage orientation at 135° with the waveguide was similar to that using the rotating microwave model regarding uniform thermal and electric field distributions. Both rotating models provided good agreement between the predicted and actual values and had greater precision than the stationary model. In addition, the computational time using COMSOL in combination with MATLAB was reduced by 60% compared to COMSOL alone. Consequently, the models could assist food producers and associations in designing packaging materials to prevent leakage of the packaging compound, developing new products and applications to improve product heating uniformity, and reducing the cost and time of the research and development stage.

## 1. Introduction

Ready-to-eat sausage has become a favorite food of consumers globally [1,2] due to its convenience, microbial safety, and unique flavor. These sausages are generally reheated using a commercial microwave in a convenience store or a household; however, a limitation of microwave heating is related to its non-uniform temperature distribution [3,4], which affects food quality and safety. Overheating might reduce product quality and increase the risk of packaging compounds leaking or contaminating the food products [5], whereas underheating might cause foodborne illnesses from pathogens such as *Listeria monocytogenes* that plausibly could contaminate the sausages [6,7]. A zero detection for *L. monocytogenes* for ready-to-eat foods was required by the Codex Alimentarius Commission (CAC) for microbiological limit [8]. The temperature distribution in food can be affected by many factors including food dielectric properties, heat capacity, thermal conductivity, physical properties (dimension, shape, and density), product geometry and the location of food in the microwave, and cavity design [9,10]. However, adopting a trial-and-error approach in an experiment is neither a time- nor cost-effective strategy. One method for improvement is to develop a mathematical model to simulate the temperature distribution and to investigate the locations of cold and hot spots in the food products under microwave heating, with the aim of reducing the cost and time of the research and development stage.

Using the finite difference time domain (FDTD) and finite element method (FEM) are popular numerical methods associated with the study of microwave heating [11], with FEM perhaps being more suitable to a complicated domain than FDTD [10]. A rotating microwave model has been developed using many different commercial software programs. Geedipalli et al. [3] used ANSYS v.8 coupled with FIDAP in 3D to calculate the temperature distribution of rectangular potatoes using constant dielectric properties. However, this technique required 24 different time steps for one round, which meant a long computational time and involved complicated operations. To overcome these drawbacks, Lui et al. [4,12] combined two commercial software programs (FEMAB and PHOTO-series) based on FEM to examine thermal distribution. They analyzed the electromagnetics and heat transfer for every rotation of 30° for 1 s, which was faster than the work of Geedipalli et al. [3]. Next, Pitchai et al. [13] studied the temperature simulation of a household microwave using a 1% gellan gel cylinder in a stationary microwave model based on FDTD using the QuickWave program. The simulated temperature was in good agreement with actual temperatures, with root mean square error (RMSE) values of 0.53–4.52 °C. However, the model might be inappropriate where there is a complex domain with rotation. Therefore, Pitchai et al. [10] improved the model by using COMSOL Multiphysics^®^ interfaced with MATLAB. However, the differences between the actual and simulated temperatures of chicken nuggets and mashed potatoes produced an RMSE in the range of 4.3–26.2 °C. Yang et al. [14] integrated mechanistic-modeling and machine-learning using the COMSOL-MATLAB LiveLink^TM^ to optimize the thickness of rectangular-, conical-, and elliptical-shaped products in rotating microwave heating. The developed program showed higher efficiency at 45.9–62.1%, as compared to the parametric sweep approach from mechanistic-modeling. Most microwave model development has focused on symmetrical shapes such as gel, mashed potatoes, rectangular-shaped fish, and chicken nuggets, which might result in such models being incompatible with other applications, particularly for sausages. Furthermore, a rotating microwave model might require a long computational time and high-performance computing due to the computational complexity.

Therefore, the current research developed a three-dimensional mathematical model to simulate the temperature distribution in ready-to-eat packaged sausages heated in a stationary microwave using COMSOL, compared to a rotating microwave using COMSOL coupled with MATLAB. Specifically, the aims of this research were: (1) to simulate the temperature and electric field distributions to identify the cold and hot spots in four ready-to-eat sausages contained in a plastic package placed in a stationary versus a rotating microwave; (2) to determine the appropriate sausage orientation that optimized heating uniformity for the stationary microwave oven; and (3) to validate the developed model by comparing the actual and predicted temperatures at four selected points in the sausages using a stationary versus a rotating microwave oven.

## 2. Materials and Methods

### 2.1. Overall Work

The overall work comprised of three parts: (1) the determination of product properties; (2) the development of a computational mathematical model; and (3) the experimental validation. The dielectric properties, electrical conductivity, specific heat capacity, thermal conductivity, and density of the sausages and the plastic packaging were determined. Equations of properties as a function of temperature were declared in MATLAB version R2015a (MathWorks, Inc., Natick, MA, USA) and linked to material properties in COMSOL Multiphysics version 5.0 (COMSOL Inc., Los Angeles, CA, USA). The temperature distribution of a packaged ready-to-eat sausage was simulated using COMSOL for the stationary and rotating heating model, and COMSOL in combination with MATLAB for the rotational heating model. The simulation was conducted on a Dell workstation with 16 GB of memory and an Intel Xeon processor (E-2124 CPU@ 3.30 GHz, 3.31 GHz). The temperature simulation was validated by comparing the actual and simulated temperatures at selected points.

### 2.2. Product

Four ready-to-eat sausages packed in a plastic package (Wiener, Charoen Pokphand Foods PCL., Bangkok, Thailand) were purchased from a local grocery store in Bangkok, Thailand. The sausages were the emulsified type consisting of 68% pork, 14% water, 12% chicken meat, and 6% seasoning (monosodium glutamate and disodium 5′-ribonucleotides). The initial temperature of the sausages was approximately 14–15 °C. Types of packaging materials were examined using a spectroscopic technique (FTIR-ATR) with an FTIR Spectrometer (Nicolet iS5, Thermo Fisher Scientific, UK). The packaging film was separated into two layers: (1) a transparent film having a wavenumber close to that of polypropylene (PP); and (2) a red opaque film identified as a linear low-density polyethylene (LLDPE) structure. The dielectric constant, dielectric loss, and electrical conductivity of PP are low and close to those of LLDPE (Table 1); therefore, the PP properties were used in the mathematical model to represent the multilayer plastic packaging properties.

### 2.3. Parameter Measurement

The dielectric constant ($\varepsilon^{\prime }$) and loss factor ($\varepsilon^{\prime \prime }$) of the sausages were determined using a high-temperature coaxial probe of an ENA series network analyzer (E5063A, Keysight, Santa Rosa, CA, USA). Three calibration standards (air, short, and deionized water) [10] at 25 °C were used for analyzer calibration in the range 900–2500 MHz. The sausage samples were contained in a double-layer stainless-steel test cell (2 cm inner diameter and 3 cm height) placed on a hotplate stirrer (IKA^®^ RCT Basic, Selangor, Malaysia) and heated with silicone oil. The dielectric constant and loss factor were measured from 10 °C to 90 °C at each 5 °C increase. The dielectric constant and loss factor of the PP sheet were obtained from Gupta and Eugene [15].

The electrical conductivity (σ) is related to the dissipated power, which is converted to thermal energy in the materials. Therefore, the electrical conductivity of sausages was calculated in the range of 5–90 °C using Equation (1) in combination with the dielectric loss factor of the sausage product [16]:

(1) $$\sigma =\omega \varepsilon_{0}\varepsilon^{\prime \prime }$$

The electrical conductivity of the PP sheet was defined as 0.0011 S/m based on Kanogchaipramot et al. [17].

The specific heat ($C_{p}$) of the sausages and packaging film was measured using a differential scanning calorimeter (DSC 1, Mettler-Toledo International Inc., Greifensee, Switzerland). Weighed amounts (each approximately 10 mg) of the sausages and packaging film samples were placed into a 40 μL aluminum pan. The specific heat of the sausage and PP packaging film was measured at a heating rate of 5 °C/min over a scanning range of 5–90 °C, which was modified from Kamonpatana et al. [18].

The thermal conductivity (k) of the sausages and PP package was used as a constant value since the product did not change phase, which slightly affected the product temperature during heating [10]. The thermal conductivity was investigated using a thermal constants analyzer (TPS 2500S, Hot Disk Medical AB, Gothenburg, Sweden). A Kapton sensor probe no. 7577 was inserted into the center of the sausage sample. In addition, the package was cut into two pieces (5 cm × 5 cm) and a Kapton sensor probe no. 7280 was inserted between both pieces. The thermal conductivity was measured at 21 °C for 20 s.

The density (ρ) of the sausages in the temperature range of 5–90 °C was calculated via the Choi and Okos equations [19]. Proximate composition of the sausages was analyzed based on the contents of moisture (AOAC 950.46), lipids (AOAC 922.16), ash (AOAC 920.13), proteins (in-house method TE-CH-042 based on AOAC 981.10), and carbohydrates (in-house method TE-CH-169) [20]. The density of the PP packaging film (900 kg/m^3^) was obtained following Kanogchaipramot et al. [17].

**Table 1:** Electrical and thermal properties of the sausage product and package.

Product	Temperature (°C)	Dielectric Property		σ (S/m)	$C_{p}$ (kJ/kg °C)	ρ (kg/m^3^)	k (W/m °C)
		$\varepsilon^{\prime }$	$\varepsilon^{\prime \prime }$				
Sausage	10	27.81	13.95	2.09	3.75	1052.74	0.476
	20	29.28	15.98	2.09	3.84	1048.57	
	30	29.55	16.41	2.11	3.93	1044.41	
	40	29.22	15.84	2.15	4.02	1040.24	
	50	28.89	14.87	2.21	4.11	1036.08	
	60	29.16	14.10	2.29	4.20	1031.91	
	70	30.63	14.13	2.39	4.29	1027.75	
	80	33.90	15.56	2.51	4.38	1023.58	
	90	39.57	18.99	2.65	4.47	1019.42	
PP	10	2.4 [15]	0.001 [15]	0.001 [17]	1.67	900 [17]	0.172
	20				1.90		
	30				2.13		
	40				2.36		
	50				2.59		
	60				2.82		
	70				3.05		
	80				3.28		
	90				3.51		
LLDPE	25	2.1 [21]	0.001 [22]	0.0007 [23]	-	-	-
Air	25	1	-	0	-	-	-
Glass	25	1	-	0	-	-	-

### 2.4. Mathematical Model Development

#### 2.4.1. Geometry of Product and Household Microwave

**Figure 1:**
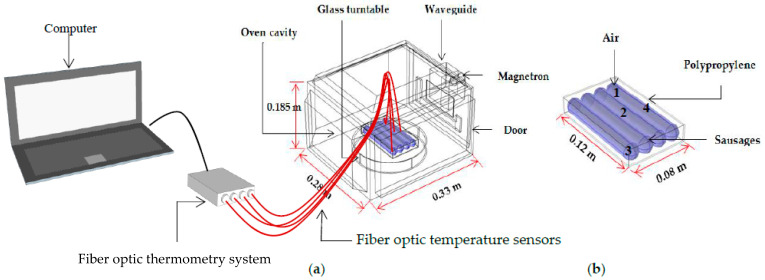
The (**a**) experimental setup of temperature measurement in household microwave and (**b**) geometry of sausages.

**Figure 2:**
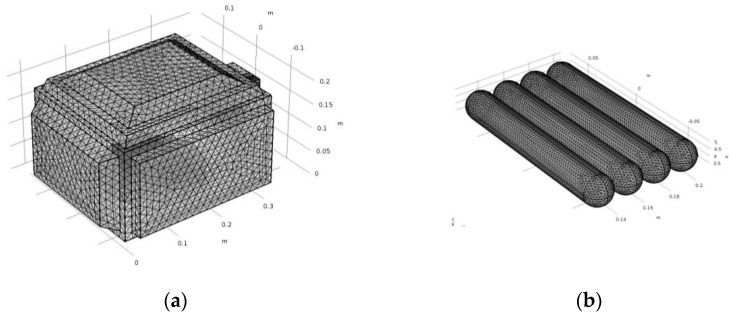
Element meshing of (**a**) microwave and (**b**) sausages domain.

The household microwave oven (ME711K, Thai Samsung Electronics Company Limited, Chon Buri, Thailand) consisted of an oven cavity of 0.33 m × 0.28 m × 0.185 m, a trapezoid waveguide, a silica glass turntable, a cylindrical magnetron, and a door. The internal heating area consisted of four sausages (0.02 m in diameter and 0.12 m in length for each piece), PP packaging film, and air. The four sausages in the package were placed at the turntable center, as shown in Figure 1. The transient temperatures of the ready-to-eat sausages at the four points were measured and recorded using fiber optic temperature sensors and a 4-channel fiber optic thermometry system (Luxtron FOT Lab kit, LumaSense Technologies, Inc., Santa Clara, CA, USA). A frequency of 2450 MHz and the coaxial feed port were used in the microwave model system in the transverse electromagnetic (TEM) mode [13]. The tetrahedral elements were automatically produced by COMSOL, as presented in Figure 2, and were manually adjusted until the change in the temperature at the selected points was not significant (*p* < 0.05; data not shown). The maximum mesh size element ($h_{max}$) of all domains was 0.017 m, while the sausage domain was 0.008 m. The mesh size elements were less than the maximum mesh size calculated using Equation (2) [24], which had a 0.01–0.04 m mesh size.

(2) $$h_{max}=\lambda /\left(6\sqrt{\varepsilon^{\prime }}\right)$$

In addition, the minimum element quality of the sausage domain obtained from COMSOL was 0.21 greater than 0.02, which was recommended by COMSOL and could be solved with fast convergence [10].

#### 2.4.2. Governing Equations

The electromagnetic heat source equations (Q) were based on the resistive loss ($Q_{rh}$) and magnetic loss ($Q_{ml}$) as demonstrated in Equation (3):

(3) $$Q=Q_{rh}+Q_{ml}$$

The resistive loss equation related to electrical properties of food materials was calculated using Equation (4):

(4) $$Q_{rh}=\frac{1}{2}\left(J\cdot E\right)$$

Magnetic loss in Equation (5) did not appear in food materials and was not used in electromagnetic heat source equations [25], since the net magnetic flux departing a region is equal to zero [26]:

(5) $$Q_{ml}=\frac{1}{2}\left(J\omega J\cdot H\right).\;$$

Therefore, the electromagnetic heat sourcwas dissipated and converted to thermal energy in food materials [11], with the power density dissipated ($P_{diss}$) according to Equation (6):

(6) $$Q=P_{diss}=\frac{1}{2}\left(J\cdot E\right)=\frac{1}{2}\omega \varepsilon_{0}\varepsilon^{\prime \prime }{\left|E\right|}^{2}$$

The electromagnetic wave equation was derived from Maxwell’s equations, where the distribution of electromagnetic in all domains was calculated using Equation (7):

(7) $$\nabla \times \mu_{r}^{-1}\left(\nabla \times \vec{E}\right)-k_{0}^{2}\left(\varepsilon_{r}-\frac{J\sigma }{\omega_{0}}\right)\vec{E}=0$$

Heat transfer in a solid was used for the sausages and packaging film domains with conductive heat transfer in the solid determined using Equation (8):

(8) $$\rho C_{p}\frac{\partial T}{\partial t}+\rho C_{p}u\cdot \nabla T=\nabla \cdot \left(k\nabla T\right)+Q$$

#### 2.4.3. Boundary Equations

A perfect electric conductor was assumed to be zero, since the electric field propagating in the boundary of the oven and waveguide was equal to zero [3,4,12], as shown in Equation (9):

(9) n×E=0

Thermal insulation was specified for the package due to negligible conductive heat transfer, as given in Equation (10):

(10) k∇T·n=0

Convective heat transfer was considered in the surface of the sausages, as shown in Equation (11):

(11) $$k\nabla T\cdot n=h\left(T-T_{ext}\right)$$

The natural heat transfer coefficient was based on Pitchai et al. [10].

### 2.5. Simulation Development

#### 2.5.1. Stationary Microwave Heating System

The sausage, PP packaging film, air in the cavity, waveguide, and glass plate were domains used in the electromagnetic and heat transfer modules in COMSOL. A commercial software program based on FEM simulated the temperature distribution at each orientation (0°, 45°, 90°, and 135° with the waveguide) for 50 s.

#### 2.5.2. Rotating Microwave Heating System

The ready-to-eat sausage was heated under counter-clockwise microwave rotation. The crucial parameters were the period time (T) used for the calculation of angular speed (ω), as depicted in Equation (12):

(12) $$\omega =\frac{2\pi }{T}$$

The average time was 12.23 s, leading to an angular speed of 29.45° s^−1^. The movement step was calculated using Equation (13), in which the time for a movement of 45° was 1.53 s.

(13) $$t=\frac{angle}{\omega }$$

#### 2.5.3. Rotating Ready-to-Eat Sausage Using COMSOL

**Figure 3:**
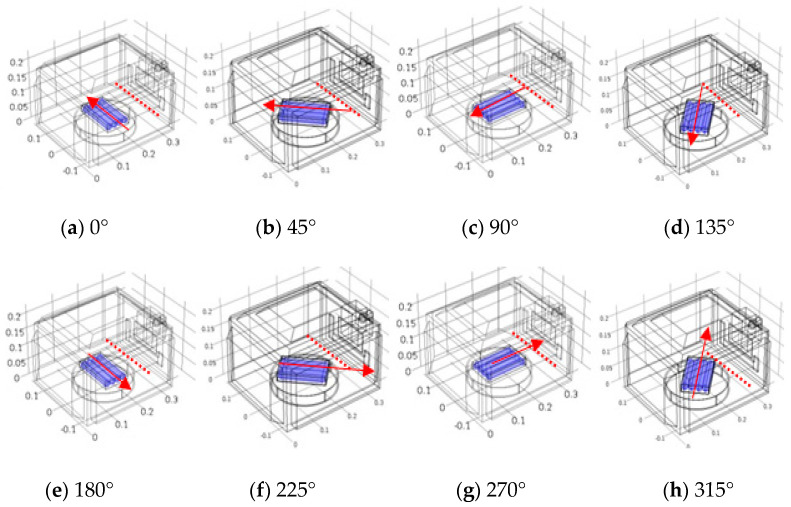
Geometry of household-microwave oven and ready-to-eat sausages at the position angle from 0° to 315° with waveguide (red dotted line).

The stationary sausage samples were placed at the center of the microwave at orientation angles with the waveguide of 0°, 45°, 90°, 135°, 180°, 270°, and 315°, as shown in Figure 3. The first orientation was assigned to be 0° with the waveguide (with an initial temperature of 15 °C) and moved for 1.53 s for every 45°. The final temperature of each 45° orientation was used as the initial temperature of the next orientation. The temperature simulation was calculated at 700 W for 60 s and analyzed using electromagnetics and heat transfer for forty-time steps (8 steps × 5 rounds). Temperature simulation using COMSOL alone, along with the heating process of 60 s (5 rounds), consumed a computational time of 22 h 34 min 50 s (Table 2).

#### 2.5.4. Rotating Ready-to-Eat Sausage Using COMSOL in Combination with MATLAB

**Figure 4:**
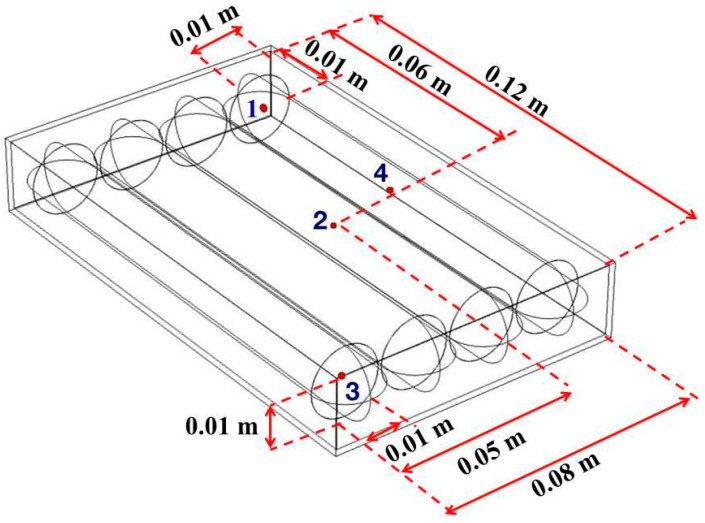
Four measurement points (points 1–4) for sausages in package.

**Figure 5:**
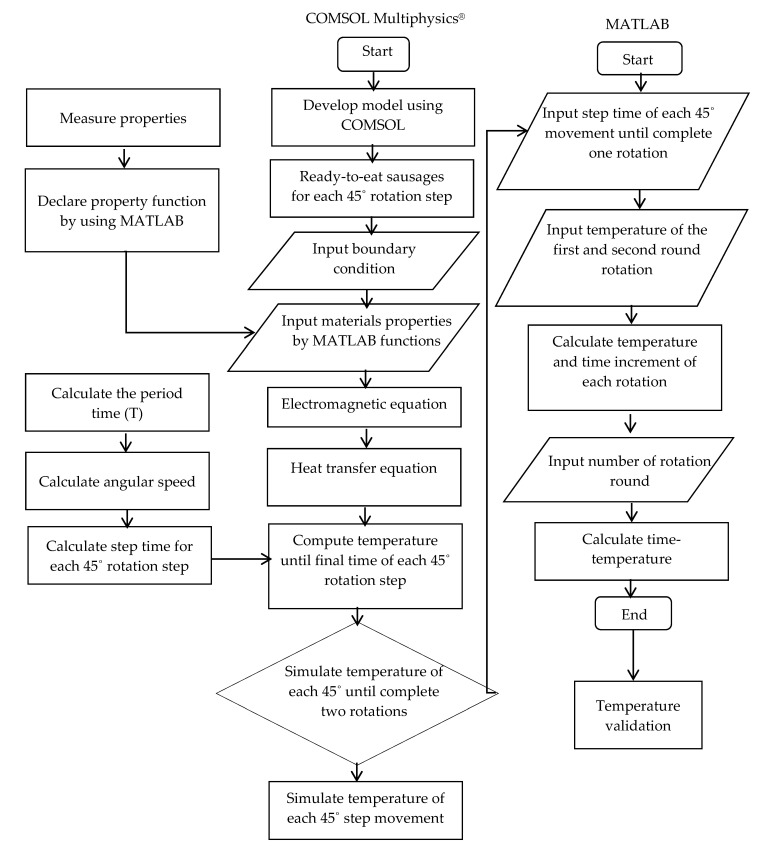
Flow diagram of COMSOL Multiphysics^®^ software in combination with MATLAB.

The rotating microwave model was developed using two commercial programs (COMSOL Multiphysics software and the MATLAB program) to calculate the temperature profile of the specific points (points 1–4, as shown in Figure 4). For the first study, the temperature distribution of sausages within the plastic package was simulated at the first and the second rotation round using COMSOL. The temperature simulation procedure is summarized in Figure 5. Temperature simulation was analyzed using COMSOL for 16-time steps (8 steps × 2 rounds), consuming a computational time of 9 h 1 min 20 s (Table 2). The temperature difference of the first and the second round was assigned in MATLAB. A temperature for each rotation was calculated to produce a temperature increase for every rotation round with increasing time. After that, the required rotation round was put in the command window. Then, the temperature-time data were obtained within 2 s based on written code commands, which was easy and fast.

The temperature of the ready-to-eat sausages was measured at points 1–4, as presented in Figure 4. The validation was conducted by comparing the simulated temperature with the measured temperature using fiber optic temperature sensors.

### 2.6. Comparison between Stationary Heating and Rotational Heating of Sausages

The package of four ready-to-eat sausages was heated at 700 W for 50 s in a stationary or a rotating microwave oven. The temperature distributions of the sausages using the stationary microwave model at the individual orientations of 0°, 45°, 90°, and 135° were compared with those for the rotating microwave model.

To investigate a reduction of *L. monocytogenes* in ready-to-eat sausages under microwave heating, the pasteurization value (PU) was calculated using Equation (14).

(14) $$\mathrm{PU}=\int_{0}^{t}10^{\frac{T-T_{ref}}{Z}}dt$$

where *t* is the heating time (min), *T* is the sausage temperature, $T_{ref}\;$is the reference temperature of 60 °C, and Z is the Z-value of 8.9 °C. The D-value at 60 °C was 1.30 min [27].

### 2.7. Statistic Analysis

The actual temperatures of the ready-to-eat sausages at the four points were measured using fiber optic temperature sensors under microwave heating in triplicate. The accuracy of the temperature simulation was analyzed using the root mean squared error (RMSE) [10,28,29], and the relative root mean square error (RRMSE) [4,12], as presented in Equation (15) and Equation (16), respectively:

(15) $$\mathrm{RMSE}=\sqrt{\frac{1}{n}\sum_{i=1}^{n}{\left(T_{p}-T_{0}\right)}^{2}}=\sqrt{\frac{1}{n}\sum_{i=1}^{n}{\left(T_{r}-T_{s}\right)}^{2}}$$

(16) $$\mathrm{RRMSE}=\sqrt{\frac{1}{n}\sum_{i=1}^{n}{\left[\left(T_{p}-T_{0}\right)/T_{0}\right]}^{2}\;}\times 100$$

In deciding on the optimal orientation of Section 2.6, we chose the minimum RMSE.

## 3. Results and Discussion

### 3.1. Thermal and Electrical Properties

The dielectric properties, consisting of the dielectric constant and the loss factor of the ready-to-eat sausages in the range 10–90 °C, are presented in Table 1. During the temperature increase from 10 °C to 70 °C, the dielectric properties slightly increased. After the sausage temperature reached 80 °C, the dielectric properties rapidly increased because the myofibril protein of the emulsion sausages may have shrunk and denatured and subsequently released water and salt [30]. The increase in the water and sodium ion (Na+) concentrations (related to dipole rotation and ionic polarization) raised the dielectric constant and loss factor [31]. The dielectric properties of the PP sheet were kept constant for the calculation and the values of the dielectric constant and loss factor were 2.4 and 0.001, respectively [15].

The electrical conductivity of the ready-to-eat sausages depended on the dielectric loss factor at a frequency of 2450 MHz in the range 10–90 °C, as shown in Equation (1). The electrical conductivity of the sausages remained relatively steady at temperature increments between 10 °C and 75 °C. However, above 80 °C, the electrical conductivity of the sausage increased due to water evaporation. Myofibril protein of emulsion sausages shrunk and denatured [30]. The concentration of sodium ion (Na+) subsequently increased, which related to the increase in dielectric loss factor and electrical conductivity. The electrical conductivity of the sausages, with varying temperatures, is depicted in Table 1. The electrical conductivity of PP was 0.0011 S/m, according to the study of Kanogchaipramot et al. [17].

The proximate analysis indicated that the sausages were composed of 55.67% moisture content, 15.53% protein, 25.75% lipid, 3.55% carbohydrate, and 2.08% ash. The density of sausages decreased with temperature increase, as shown in Table 1, whereas that of the PP package was 900 kg/m^3^, obtained from the study of Kanogchaipramot et al. [17].

The specific heat and thermal conductivity of the sausages and packages are reported in Table 1. The specific heat of the sausage slightly increased with temperature (3.75 kJ/kg °C at 10 °C and 4.47 kJ/kg °C at 90 °C). The thermal conductivity values of the sausage and PP were 0.476 and 0.172 W/m °C, respectively. Since thermal conductivity slightly affects temperature changes [17], the thermal conductivity could be a constant value in the mathematical model [10].

### 3.2. Experimental Validation

#### 3.2.1. Stationary Microwaves

**Figure 6:**
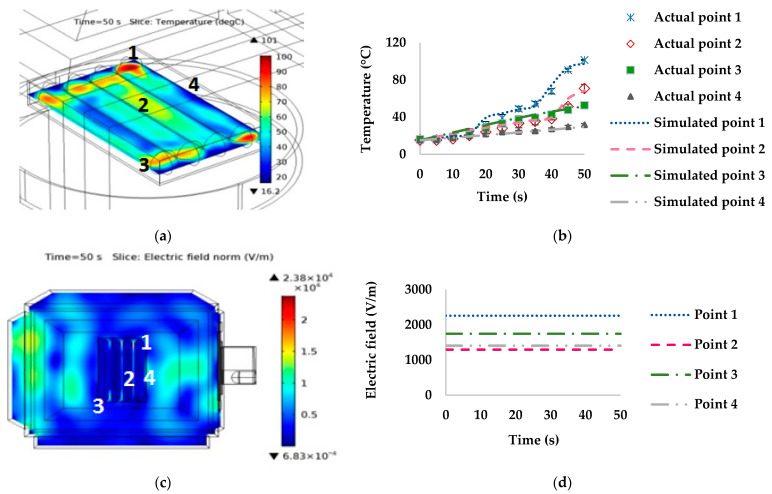
Effect of simulated stationary microwave on (**a**) thermal simulation, (**b**) temperature profile at points 1–4, (**c**) electric field, and (**d**) electric field profiles at points 1–4 of ready-to-eat sausage at 0° orientation to waveguide.

The simulated and measured temperatures at points 1–4 and a 0° orientation with the waveguide are presented in Figure 6. Point 1 had the highest temperature (measured and simulated temperatures of 101.38 °C and 97.38 °C, respectively) at 50 s. In contrast, point 4 had the lowest temperatures (measured and simulated temperatures of 31.67 °C and 29.44 °C, respectively). The simulated temperature distribution (Figure 6a,b), indicated that the hot spot was approximately 100 °C, while the cold spot was approximately 30 °C. The highest electric field distribution was at point 1, whereas at points 2 and 4 it was lowest, as presented in Figure 6c,d, respectively.

**Figure 7:**
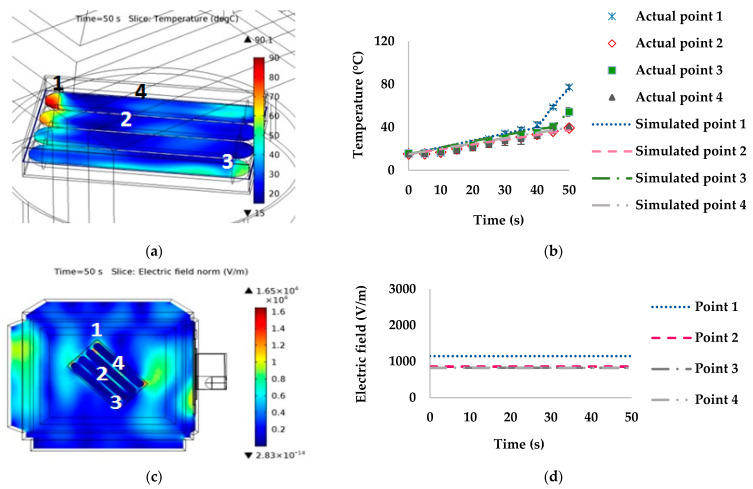
Effect of simulated stationary microwave on (**a**) thermal simulation, (**b**) temperature profile at points 1–4, (**c**) electric field, and (**d**) electric field profiles at points 1–4 of ready-to-eat sausage at 45° orientation to waveguide.

The temperature and electric field at points 1–4 and a 45° orientation with the waveguide were lower than at 0°, as illustrated in Figure 7. The temperature at point 1 during 41–50 s rapidly increased, with the measured and simulated temperatures at 50 s being 82.67 °C and 76.28 °C, respectively. Points 2 and 4 had the lowest temperatures (measured temperature of 39.33 °C at point 2 and 40.41 °C at point 4). In contrast, the simulated temperatures were 37.27 °C at point 2 and 39.81 °C at point 4. The electric field at points 1–4 (Figure 7c,d), corresponded with the temperature of points 1–4, with point 1 having the highest values for both temperature and electric field. The difference between the hot and cold spots was approximately 55 °C.

**Figure 8:**
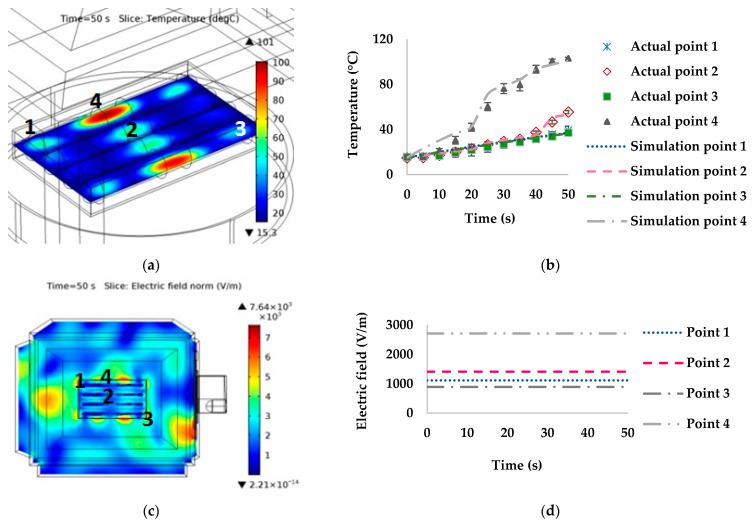
Effect of simulated stationary microwave on (**a**) thermal simulation, (**b**) temperature profile at points 1–4, (**c**) electric field, and (**d**) electric field profiles at points 1–4 of ready-to-eat sausage at 90° orientation to waveguide.

When the orientation changed to 90°, point 4 had the highest temperature and electric field strength, while points 1 and 3 had the lowest temperatures and electric field strengths, as illustrated in Figure 8. The temperature distribution indicated that the hot spot area corresponded with point 4 (with simulated and measured temperatures at 50 s of 103.00 °C and 100.96 °C, respectively). On the other hand, the cold spot areas had a simulated temperature of approximately 30 °C.

**Figure 9:**
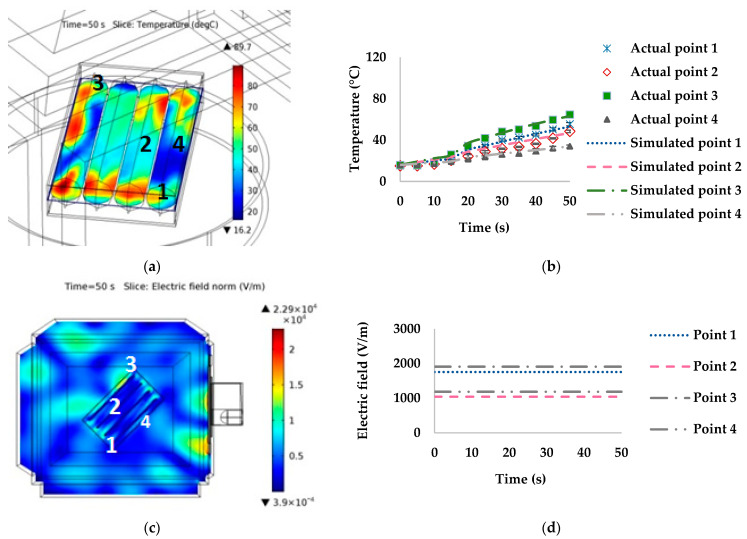
Effect of simulated stationary microwave on (**a**) thermal simulation, (**b**) temperature profile at points 1–4, (**c**) electric field, and (**d**) electric field profiles at points 1–4 of ready-to-eat sausage at 135° orientation to waveguide.

At 135° orientation with the waveguide (Figure 9), the highest temperature was at point 3 (with measured and simulated temperatures at 50 s of 64.47 °C and 63.15 °C, respectively). The lowest temperature was at point 4 (with measured and simulated temperatures at the 50 s of 31.81 °C and 33.83 °C, respectively). The difference between the hot and cold spots was 55 °C.

The temperature distributions of sausages in the stationary microwave heating model were non-uniform. There was a rapid increase in temperature in some areas of the sausages due to the high electric field, heat accumulation from microwave heating, chemical composition changes, proteins denaturation, and thermal and moisture accumulation in the package. The high electric field at the sausage tip might have contributed to the rapid increase in temperature [32]. For example, at point 1 for orientations 0° and 45°, the electric field values were 2257.10 and 1141.90 V/m, respectively (Figure 6 and Figure 7). Point 4 at 90° had the highest temperature related to the electric field of 2711.90 V/m (Figure 8). On the other hand, a low temperature was linked to the low electric field, such as at point 4 at orientations of 0° (Figure 6) and 45° (Figure 7), at point 3 at 90° (Figure 8), and at point 4 at 135° (Figure 9). 

The temperature of the heated sausages rapidly increased, probably due to the change in chemical compositions of the sausages and protein denaturation. Myofibril proteins were solubilized by sodium chloride, which subsequently encapsulated fat droplets and bound water to form the gel matrix of the sausage emulsion [31]. When the temperature slowly increased until reaching 45–50 °C, the myofibrils and sarcoplasmic proteins began to shrink [33], and the structure of the sausages changed, accounting for the dispersion of water, and ionic compounds, which were dispersed over the gel matrix structure [30]. The effect of water and ionic compounds resulted in the rapid acceleration of dipole rotation and ionic polarization in microwave heating, leading to a rapid increase in temperature [34]. There was a slow increase in the temperature at point 1 with 0° orientation and at point 4 with 90° orientation, until the temperature reached approximately 45–50 °C; then it sharply increased and subsequently remained stable at 100 °C. It was observed that the shrinkage occurred at 45 °C for myofibrils and at 40–50 °C for sarcoplasmic protein [28], resulting in water dispersion and salt in the sausages.

The ranges of RMSE and RRMSE were 1.06–4.05 °C and 4.91–20.26%, respectively, in the stationary microwave heating model of the sausages at orientations of 0°, 45°, 90°, and 135°. The value of RMSE was acceptable for validating the microwave heating model [10,28,29], whereas there were high values for RRMSE that may be due to the greater sensitivity of RRMSE than of RMSE. In addition, the errors might be attributed to actual thermal and moisture accumulation, protein denaturation, and fiber optic sensor movement.

#### 3.2.2. Rotating Microwaves

**Figure 10:**
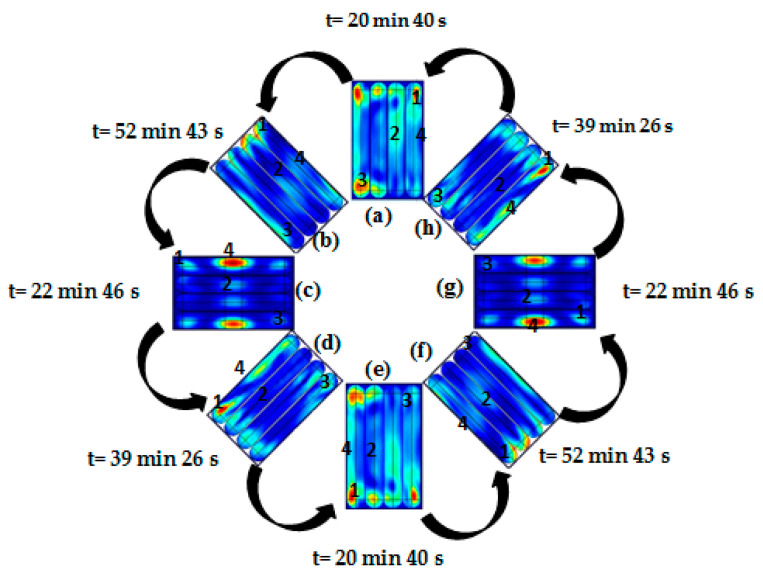
Thermal simulation of rotating ready-to-eat sausage under microwave heating at angle position of (**a**) 0°, (**b**) 45°, (**c**) 90°, (**d**) 135°, (**e**) 180°, (**f**) 225°, (**g**) 270°, and (**h**) 315°, simulated by using COMSOL.

**Figure 11:**
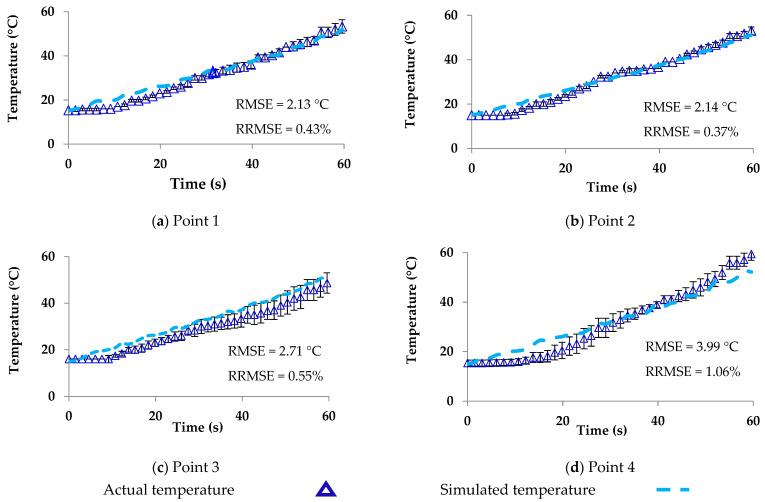
Actual and simulated temperature of ready-to-eat sausage under microwave rotating at points 1–4 from COMSOL.

**Figure 12:**
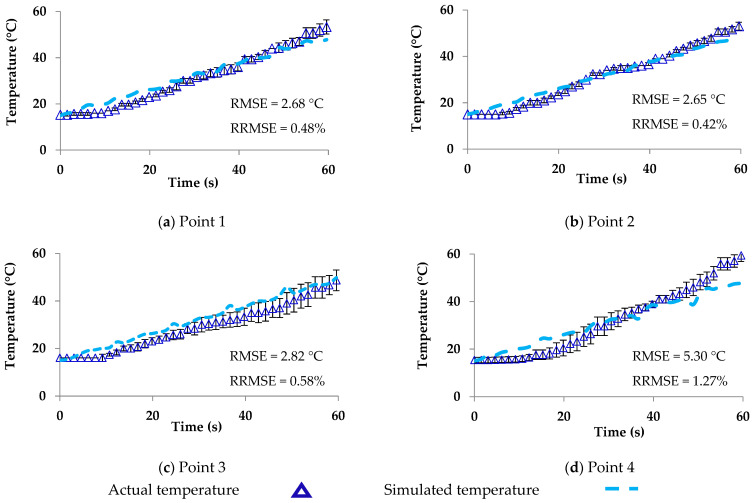
Actual and simulated temperature of ready-to-eat sausage under microwave rotating at points 1–4 from COMSOL in combination with MATLAB.

The temperature distributions in one cycle rotation at rotation angles of 0°, 45°, 90°, 135°, 180°, 225°, 270°, and 315° with the waveguide are shown in Figure 10. The simulated temperatures (points 1–4) from COMSOL and from COMSOL in combination with MATLAB were compared to the measured temperatures reported in Figure 11 and Figure 12, respectively. The ranges of RMSE and RRMSE for all four points from COMSOL were 2.13–3.99 °C and 0.37–1.06%, respectively, while for COMSOL in combination with MATLAB, they were 2.65–5.30 °C and 0.42–1.27%, respectively. Both simulations indicated good agreement between the measured and predicted temperatures. The computational time using COMSOL alone was 22 h, 34 min, and 50 s for five rotating rounds (Table 2). On the other hand, COMSOL in combination with MATLAB reduced the computational time to 9 h, 1 min, and 26 s (Table 2). Therefore, COMSOL in combination with MATLAB could decrease the total computational time by 13 h, 33 min, and 24 s or by 60%. In addition, the MATLAB coding led to many advantages, such as flexible simulation, rapid calculation, and reduced time, cost, and waste of materials during the product development and package design.

### 3.3. Comparison between Stationary and Rotating Models

The temperature distribution profiles between the stationary heating model at orientations of 0°, 45°, 90°, and 135° with the waveguide and the rotating heating model were compared to examine the appropriate orientation of the sausages heated in the stationary microwave. The RMSE values between of the stationary and rotating models were 14.35, 6.98, 18.38, and 5.04 for 0°, 45°, 90°, and 135°, respectively, The temperatures from the stationary microwave heating model at 135° were close to those from the rotating microwave heating model with the lowest value of the RMSE; and were more uniform than for the other orientations. Therefore, orientation at 135° with the waveguide for the packaged sausages was suggested for a stationary microwave to obtain more uniform heating compared to other orientations.

The difference between cold spot and hot spot temperatures for 0°, 45°, 90°, and 135° at the heating time of 50 s was 69.71, 43.34, 66.00, and 30.66 °C, respectively, while that for rotating microwave heating was 7.38 °C (Table 3). Rotating microwave heating provided more uniform heating than stationary microwave heating. Since PU was linked to product temperature, the difference between the minimum and maximum PU of stationary microwave heating was high and the minimum PU was 0–0.001 min. For the rotating microwave heating, more uniform PU were observed and the minimum PU was 0.001 min. At the heating time of 50 s, the minimum PU was low and might not reduce the number of microorganisms. However, due to uniform heating, a rotating microwave heating could increase the heating time to 60 s and 70 s, and obtain the minimum PU of 0.006 and 0.092 min, respectively. To reduce the contamination counts of *L. monocytogenes* from <10–100 CFU/g [35] to the zero detection limit [36], the increase in heating time is required. However, product quality should be considered. The simulations can assist food producers to ensure food safety and maintain product quality. 

## 4. Conclusions

Electromagnetic heat source, electromagnetic wave, and heat transfer equations were solved to simulate the temperature distribution and to indicate hot and cold spots within four sausages contained in a plastic package. Orientation was the crucial factor that affected the temperature distribution of the packaged sausages. The sausages at an orientation of 135° with the waveguide had greater heating uniformity than the sausages at other orientations, and their temperature was comparable to those using the rotating microwave oven. Therefore, 135° was recommended for heating packaged sausages in a stationary microwave oven.

For a rotating model, the model using COMSOL in combination MATLAB reduced the computational time by 60% of that required when using COMSOL alone. The values for the RMSE and RRMSE at four points in the packaged sausages for the rotating microwave model using COMSOL alone were 2.13–3.99 °C and 0.37–1.06%, respectively, while using COMSOL and MATLAB they were 2.65–5.30 °C and 0.42–1.27%, respectively. The two rotating microwave models were in good agreement regarding the measured and simulated temperatures of the packaged sausages. The models showed that the interactions of food and microwave improved heating uniformity and the models predicted the hot spot temperature for consideration in using packaging materials. In addition, the models were flexible, easy, and fast and could be applied for different food types in microwave ovens to assist food producers and associations in the research and development of new products and applications.

## Figures and Tables

**Table 2:** Computational time of temperature simulation of COMSOL and COMSOL in combination with MATLAB (COMSOL and MATLAB).

Rotation Round	Computational Time	
	COMSOL	COMSOL and MATLAB
1 (steps 1–8)	4 h 31 min 10 s	4 h 31 min 10 s
2 (steps 9–16)	9 h 1 min 20 s	9 h 1 min 20 s
3 (steps 17–24)	13 h 32 min 30 s	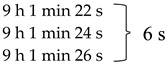
4 (steps 25–32)	18 h 3 min 40 s	
5 (steps 33–40)	22 h 34 min 50 s	
Total times	22 h 34 min 50 s	9 h 1 min 26 s

**Table 3:** Comparison of PU between stationary and rotating microwave heating.

**Point**	**Stationary Microwave Heating**							
	**Time = 50 s**		**Time = 50 s**		**Time = 50 s**		**Time = 50 s**	
	**0°**		**45°**		**90°**		**135°**	
	**Temperature (°C)**	**PU** **(min)**	**Temperature (°C)**	**PU** **(min)**	**Temperature (°C)**	**PU** **(min)**	**Temperature (°C)**	**PU** **(min)**
1	101.38	3940.153	82.67	29.412	39.28	0.001	55.33	0.035
2	70.79	1.370	39.33	0.001	55.35	0.028	48.33	0.005
3	52.66	0.018	54.32	0.020	37.00	0.000	64.47	0.363
4	31.67	0.000	40.41	0.001	103.00	9303.928	33.81	0.000
**Point**	**Rotating microwave heating**							
	**Time = 50 s**			**Time = 60 s**		**Time = 70 s**		
	**Temperature ** **(** **°** **C)**	**PU ** **(min)**		**Temperature ** **(** **°** **C)**	**PU ** **(min)**	**Temperature ** **(** **°** **C)**	**PU ** **(min)**	
1	46.33	0.003		54.00	0.021	63.00	0.180	
2	46.67	0.003		53.67	0.020	58.00	0.092	
3	42.00	0.001		50.00	0.006	65.33	0.242	
4	49.38	0.005		61.81	0.097	70.13	1.597	

## Data Availability

Data is contained within the article.

## Abbreviation

ε	permittivity
$\varepsilon^{\prime }$	dielectric constant
$\varepsilon^{\prime \prime }$	dielectric loss factor
$\varepsilon_{r}$	relative permittivity
f	frequency, Hz
$C_{p}$	specific heat transfer, kJ/kg°C
k	thermal conductivity, W/m°C
ρ	density, kg/m^3^
λ	wavelength, m
*C*	speed of light, 3 × 10^8^ m
E	electric field, V/m
H	magnetic field, A/m
J	electric current density vector, A/m^2^
ω	angular frequency, rad/s
μ	permeability, H/m
$\mu_{r}$	relative permeability
$k_{0}$	wave number
σ	electrical conductivity, S/m
u	velocity, m/s
Q	volumetric rate of heating, W/m^3^
$p_{diss}$	power density dissipated, W/m^3^
n	unit normal vector
*h*	heat transfer coefficient, W/m^2^ °C
T	period time, s
t	time, s
$\varepsilon_{0}$	permittivity of free space, 8.854 × 1012 F/m
$E_{rmr}$	root mean square (average) electric field, V/m
$Q_{rh}$	loss of resistance, W/m^3^
$Q_{ml}$	loss of electromagnetics, W/m^3^
$h_{max}$	maximum mesh size element, mm
RMSE	root mean squared error, °C
RRMSE	relative root mean squared error, %
$T_{ext}$	external temperature, °C
$T_{0}$	experimental temperature, °C
$T_{p}$	predicted temperature, °C
$T_{r}$	temperature from rotating microwave model, °C
$T_{s}$	temperature from stationary microwave model, °C
